# Inhibitory Control and Craving in Dual Disorders and Recurrent Substance Use. Preliminary Findings

**DOI:** 10.3389/fpsyt.2021.569817

**Published:** 2021-02-03

**Authors:** Judith C. L. M. Beerten-Duijkers, Constance Th. W. M. Vissers, Mike Rinck, Jos I. M. Egger

**Affiliations:** ^1^Donders Institute for Brain, Cognition and Behaviour, Radboud University Nijmegen, Nijmegen, Netherlands; ^2^Centre of Excellence for Korsakoff and Alcohol Related Cognitive Dysfunctions/Addiction Care, Vincent van Gogh Institute for Psychiatry, Venray, Netherlands; ^3^Kentalis Academy, Royal Dutch Kentalis, Sint-Michielsgestel, Netherlands; ^4^Behavioural Science Institute, Radboud University, Nijmegen, Netherlands; ^5^Centre of Excellence for Neuropsychiatry, Vincent van Gogh Institute for Psychiatry, Venray, Netherlands; ^6^Stevig Specialized and Forensic Care for People With Intellectual Disabilities, Dichterbij, Oostrum, Netherlands

**Keywords:** dual diagnosis, substance use disorder, event related potentials, P300, N200, executive function, impulsivity, dual disorders

## Abstract

**Objective:** In Dual Disorders (DD), which involves the co-occurrence of a disorder in substance use and a mental disorder, recurrent struggles with addictive behavior are frequent. Neuropsychological knowledge concerning the profile of inhibitory control and the irresistible urge to use substances (craving) within the DD patient group may contribute to the prevention of this recurrent addictive behavior.

**Methods:** Inhibitory control and craving were assessed in 25 patients with DD and 25 healthy controls (HC). Inhibitory control tasks (Go/No-go task and Stop Signal Task) were performed combined with brain measurements (Event Related Potentials) mapping inhibitory control. Moreover, implicit and explicit measures concerning craving were administered. Statistical DD and HC comparisons, correlational and regression analyses on exploratory base were conducted.

**Results:** DD patients committed more inhibitory control errors than HC when confronted with (alcohol) consumption-related picture stimuli. Furthermore, patients with DD showed higher levels of implicit and explicit craving. The number of inhibitory control errors was positively related to levels of implicit and explicit craving. Moreover, explicit craving and impulsivity (as a dimension of inhibitory control) predicted the severity of addictive behavior. Event Related Potential analyses did not show differences in inhibitory control-associated brain activity between DD patients and HC; both groups showed reduction of P300 amplitudes in response to alcohol pictures.

**Conclusions:** Impulsivity and craving are elevated in DD patients and show predictive value for the severity of addictive behavior. One's level of impulsive action tendency may trigger less effort to control (recurrent) substance use. The findings may contribute to existing DD treatment indications by the promotion of impulse control training via “stop-think-act” methods for DD patients.

## Introduction

Failures to stop unhealthy behavior like substance use, and an irresistible urge for that use, seem central to addiction. Addiction can be described as an “enduring, inordinately strong tendency to engage in some form of pleasure producing or pain reducing behavior in a pattern that is characterized by (1) recurrent failures to *control* the behavior, and (2) *continuation* of the behavior despite significant harmful consequences” [(1), p. 270]. A better understanding of recurrent substance use behavior in addiction can be accomplished by analyzing it from the neuropsychological brain-behavior perspective. The brain-behavior model describes how brain processes direct and interact with neurocognitive functions and how these functions in turn direct and interact with human behavior (like substance use), while contextual factors can be of facilitating or disruptive influence on these brain processes, neurocognitive functions and behavior (2, 3).

The stopping failure in addiction and the mentioned urge to use substances, are respectively subsumed under the interrelated concepts of inhibitory control and craving (4). These concepts to date illustrate how patients that struggle with an addiction can perseverate in vicious circles of unhealthy behavior [see a review from (5)]. Recurrent substance use may count even more for patients with Dual Disorders (DD), which always involve a disorder in substance use, co-occurring with another mental disorder. The World Association on Dual Disorders advocates the term Dual Disorders. Another regularly used term with the same definition is Dual Diagnosis. DD combinations can have a shared cause, or can cause/intensify each other's utterance. For example, in many combat veterans a DD combination is present of a Post-traumatic Stress Disorder and a disorder in substance use. This combination may worsen the utterance of unhealthy substance use behavior (6). Higher levels of mood and temperament problems like hypomanic symptomatology correlate with higher co-morbid levels of substance use disorders, and with higher impulsivity and sensation seeking tendencies (7, 8).

Inhibitory control is a broad construct that encompasses biological (brain), cognitive, behavioral, and contextual aspects (4, 9–12). Deficits in inhibitory control are also seen as a reflection of impulsive action (13). In addition, on the brain level, inhibitory control and craving show neurobiological interrelatedness. Inhibitory control is an essential part of a Stop system in prefrontal and subcortical brain circuitry, which executes control by suppression of undesired responses. Whereas craving could be driven by a prefrontal cortex Go system which let's one engage in habits that can elicit an increase in dopaminergic effects (4). Because of the multifaceted character of inhibitory control and craving, assessment via multiple (both explicit or implicit) methods is the preferred strategy. Moreover, the use of multiple methods lines up the entire neuropsychological brain-behavior model (2, 3). Operationalization can be realized by use of neurobehavioral and electrophysiological instruments as well as through self-report.

As to the electrophysiological measurement of inhibitory control, several studies have demonstrated that the Event Related Potential (ERP) technique is particularly valuable for bringing to light perception and attention processes (14). In particular, N200 and P300 ERP components have been associated with inhibitory control, and were studied thoroughly in substance use disorder populations [for a systematic review of the literature see (15)]. The N200 component is described as negative going amplitude, occurring approximately 200 milliseconds after the onset of a low-probability No-go-target within a highly probable Go-target context. The P300 component is described as a positive going amplitude peak that in turn occurs approximately 300 milliseconds after stimulus onset of a low-probability No-go-target (16, 17). Reduction of N200 and P300 amplitudes, have been shown for disorders in substance use, however, they are inconsistent for P300 amplitudes (most frequently assessed in alcohol use studies), and they are generally associated with behavioral dis-inhibition/poor inhibitory control (e.g., in No-go tasks) (15, 18–22). In the current study, a Go/No-go task is used to measure inhibitory control aspects, using a combination of alcohol related stimuli vs. non-alcohol related stimuli.

Since a substantial percentage of patients with SUD/DD show recurrent periods of substance use despite treatment programs, continuation of the search for a better understanding and control over factors that are involved in recurrent substance use remains relevant. So far, the number of studies that addressed inhibitory control and/or craving in a multi-method manner within a DD group of patients is limited, when compared to the number of studies in groups of patients that are solely described as having a disorder in substance use (23, 24).

The present study aims to contribute to the neuropsychological understanding of DD patients' persistent struggle with recurrent substance use behavior, by zooming into the neurocognitive function of inhibitory control and craving from a multi-method perspective, including both electrophysiological, neurobehavioral and self-report measures.

All of the above leads to the following main question of this study: do patients with DD, suffering from recurrent addictive behavior, show deficits in inhibitory control and/or show higher levels of perceived craving, as compared to healthy controls (HC)? And as a sub-question: are the levels of functioning on inhibitory control and craving interrelated, possibly also strengthening the severity of addictive (drinking) behavior?

Firstly, it is hypothesized that patients with DD indeed show more problems on inhibitory control measures and higher levels of experienced craving, as compared to HC. A large effect is expected on this part (4). Secondly, concerning the N200 and P300 ERP components as associated with inhibitory control, reduction of mean activity is expected for the DD patient group as compared to HC. That is, reduction of mean N200 and P300 activity is a sign for impairments in inhibitory control (15). Thirdly, as inhibitory control and craving show neurobiological interrelatedness in addiction (4) it is hypothesized that levels of experienced implicit and explicit craving in DD will show positive correlations with the amount of inhibitory control errors in DD. Thus: a higher amount of craving is expected to be related to a higher number of inhibitory control errors on a Go/No-go task involving addiction-related pictures. The fourth hypothesis states that the interaction of inhibitory control and craving has a predictive value for the classified severity of addictive (drinking) behavior. Thus: the more inhibitory control errors one commits, and the more craving one experiences, the more severe one's daily life addictive behavior will be, classified on a substance use disorder identification test.

## Materials and Methods

### Participants

Based on a power calculation [with G^*^Power 3.1; (25)] with an estimated effect size of d = 0.50, power of 1-ß = 0.80 and alpha = 0.05 for the DD and HC group comparisons, the sample sizes as recommended were 51 per group. Unfortunately, these sample sizes could not be attained within the study's data collection time frame (2014–2017) when adhering to inclusion/exclusion criteria like abstinence periods (also see procedure section below and discussion). Eventually, 25 patients with DD and 25 HC participated, fully meeting inclusion criteria. The DD group and HC group did not differ on mean age or intelligence (see Table 1). The DD group contained significantly more males than the HC group did (6 of 25 DD patients were female and 16 of 25 HC were female; *p* < 0.01). Therefore, sex was taken into account as an extra between-subjects factor in the analyses. In 23 of 25 DD participants, alcohol was a major part of the SUD (14 patients solely used alcohol as the substance of interest, nine patients used both alcohol and drugs, and two male patients solely used drugs. However, one of these two patients did earlier get into problems with alcohol use. DD's and substances of choice as present in the patient group are depicted in Table 2.

**Table 1:** Descriptive statistics (Means and standard deviations for age, non-verbal intelligence screening, verbal intelligence screening) for dual disorders and healthy control groups.

**Measure**	**Age**		**Verbal intelligence**		**Non-verbal intelligence**	
**Group**	**Mean**	**SD**	**Mean**	**SD**	**Mean**	**SD**
Dual Disorders (*n* = 25)	41.80	11.97	95.20	7.90	113.70	9.09
Healthy control (*n* = 25)	40.96	13.71	99.60	10.82	117.96	8.87
Significance value	*p* = 0.82		*p* = 0.11		*p* = 0.10	

**Table 2:** Demographic statistics DD group.

**Dual Disorders and major substance of choice**	**Frequency**
Mood disorder	9
Psychotic disorder	4
Personality pathology	21
Anxiety disorder	9
Attention deficit (hyperactivity) disorder	9
Developmental disorder like Autism spectrum disorder	3
Alcohol	14
Cannabis	1
Alcohol and cannabis	4
Alcohol and cocaine/speed	1
Cannabis and cocaine/speed	1
Poly use of alcohol and more than one kind of other drug	4

All participants were native speakers of Dutch, and had no neurological impairments or loss of consciousness ever in their life history. No Substance Use Disorders or other psychiatric impairments as assessed by clinical interviews (see below) were present in the HC group. All patients were substance-abstinent for a minimum of 6 weeks to prevent acute or sub-acute substance influences on cognitive functioning (26). Substance abstinence was mostly controlled for by alcohol/drug tests that were a regular part of the patients' treatments. The average substance abstinence period was 12.28 weeks (range from 6 to 32 weeks). All participants had normal or corrected-to-normal vision and sufficient reading abilities, and they showed intelligence scores of 80 or higher on both verbal and non-verbal capacity screening tasks (range 82–121 for verbal intelligence; range 96–127.5 for non-verbal intelligence). Total years of problematic substance use for the patient group ranged from 7 to 47 years and the starting age of substance use ranged from 11 to 28 years.

### Materials

The major neurobehavioral, electrophysiological and self-report measures of use in this study are visualized in Table 3.

**Table 3:** Materials as used for major paradigms.

**Paradigm**	**Task**
Inhibitory control	Go/No go task
	Stop signal task (SST)
	Barratt impulsiveness scale (BIS 11)
	event related potentials components N200 and P300
Craving	Obsessive compulsive drinking scale (OCDS)
	Alcohol urge questionnaire (AUQ)
	Alcohol-implicit association task (Alcohol-IAT)
Severity addictive behavior	Alcohol use disorder identification test

### Tasks

#### Neurobehavioral Inhibitory Control Measures

##### Go/No-Go Task

**Figure 1:**
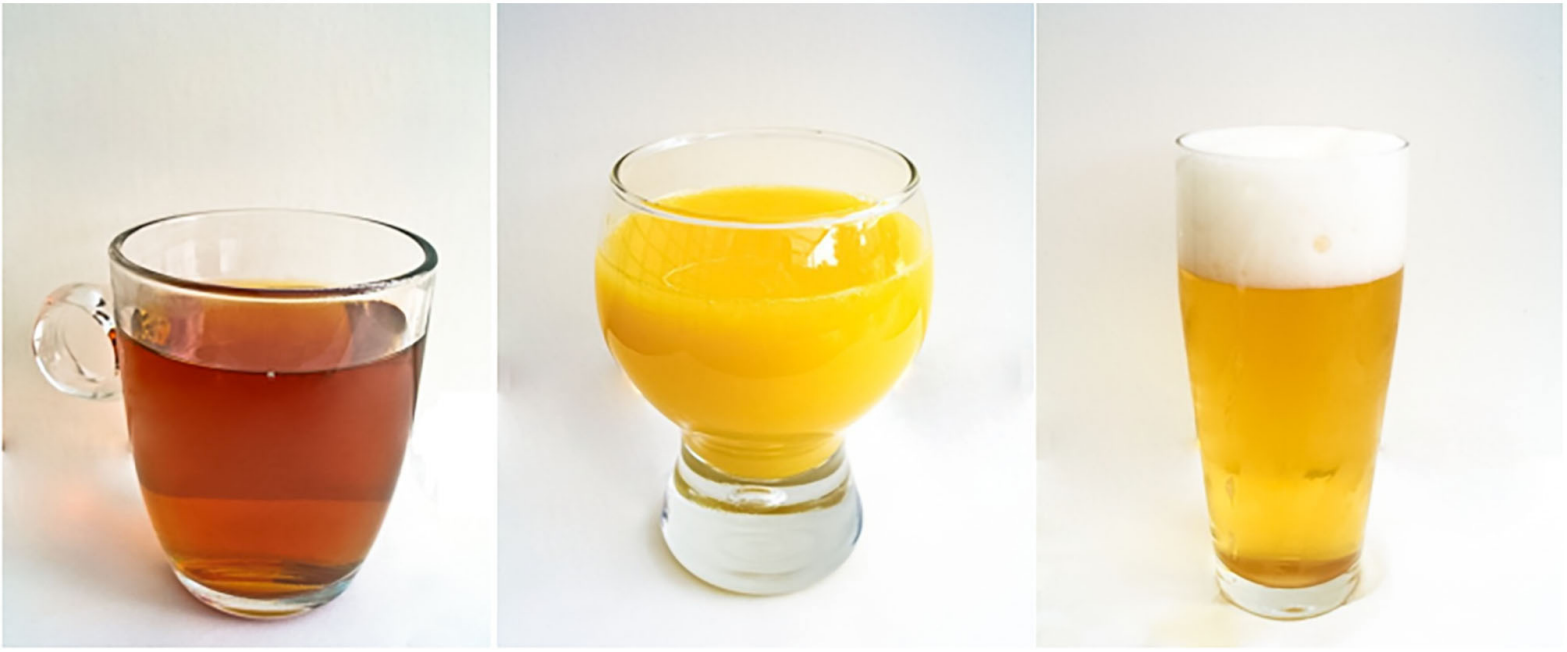
Pictures of the used Go/No-go task stimuli.

The Go/No-go task consisted of a (1) Visual standard oddball Go part (Tea picture stimuli 80%, NoGo, Beer/Orange juice picture stimuli 10% Go), and (2) No-go-part (Tea 80% Go, Beer/Orange juice 10% No-go). The task was programmed in Delphi; a Go/No-go task by Matheus-Roth and colleagues served as an example (27). Matching on aspects like hue and luminance, took place when constructing the pictures, which are depicted in Figure 1. Each of the two task conditions was preceded by practice trials of 10 picture stimuli. Pictures were presented on a computer screen for 500 milliseconds (ms.), each, against a white background (screen resolution width = 1,024 pixels/height = 768 pixels/pixel depth = 16). A small fixation cross was presented for 2,000 ms. between the picture appearances, for the subjects to look at. During the two task conditions (Go and No-go) 312 pictures were presented to the participants in a randomized order, of which 80% (250) were (non-alcoholic) tea pictures, 10% (31) were alcohol-related pictures and 10% (31) were (non-alcoholic) orange juice pictures. In the Go-part, participants were asked to react to alcohol or orange juice pictures by pressing the button box, but not to react to tea pictures. In the No-go part, they were asked to react to tea pictures by pressing the button box, but not to react to alcohol or orange juice pictures. No feedback was given during the task concerning correctness of responses. Event Related Potentials (ERPs) as brain measure of inhibitory control were recorded during the task. The task administration time was approximately 26 min. The number of commission errors (push of button when no push was required), and reaction times (time in ms. between picture appearance and button push) were used for analyses.

##### Stop Signal Task [Part of Cambridge Neuropsychological Test Automated Battery (CANTAB)]

Measuring response inhibition, this task consisted of two parts. Initially, participants were asked to press the left hand button when a left-pointing arrow was shown on the screen, and press the right hand button when a right-pointing arrow was shown on the screen. Thereafter, the participants were told to keep pressing the buttons as before, but if they heard an auditory signal (beep), they should withhold their response and not press the button (www.cantab.com). The proportion of successful stops and reaction times (time between arrow appearance and button press) were used for analyses.

#### Self-Report Inhibitory Control Dimension Measure

##### Barratt Impulsiveness Scale-Eleventh Edition, Dutch Version (BIS-11)

The Barratt Impulsiveness Scale (BIS-11) is a questionnaire measuring aspects of impulsivity, with sufficient reliability and validity. Higher item-scores are indicative of a higher amount of impulsivity (some items being reverse-scored) (28, 29).

#### Brain Measures Inhibitory Control

##### Event Related Potentials (ERP), Mean Values of N200 and P300 ERP Amplitudes

In the present study, Event Related Potentials (ERP) data were collected during the Go/No-go task, in order to observe the P300 and N200 components that are associated with inhibitory control. The N200 was defined as the mean amplitude value (uV) in the 200-300 ms. time segment after onset of the response. The P300 was defined as the mean amplitude value in the 300-450 ms. time segment after onset of the response. Furthermore, N200 and P300 components have shown to be most evident on or nearby the EEG electrode sites Fz, FCz, Cz and C4 (for N200) and FCz, Cz, C3 and C4 (for P300) [among others, (30–32)]. Based on the combination of knowledge from this past research and on visual inspection of the current data, electrode sites Fz, C4, F3, and F4 were used for the N200, and Fz, C4, F3, and F4 were used for the P300 in the present study.

#### Craving Measures

##### Drinking Identity Implicit Association Test (Drinking Identity-IAT)

Lindgren and colleagues, described the Drinking Identity IAT as “a reaction time task that requires participant to rapidly classify stimuli into superordinate categories. The strength of participants' associations between those categories is posited to be indexed by the relative speed at which they classify stimuli into categories when the categories are paired to match vs. contradict their involuntary associations between those categories. Participants were asked to classify stimuli representing two identity “target” categories (“me” and “not me”) and two drinking “attribute” categories (“drinker” and “non-drinker”). Drinker/partier/drink/ drunk suit the same category, and so do non-drinker/abstainer/sober/abstain, and me/my/mine/self and not me/they/them/theirs/other. The IAT consisted of 7 blocks, in which participants completed trials that contained single word stimuli presented in the center of the computer screen. The categories the participant could choose from for stimuli classification were at the left and right of the screen. There was no time limit for responses. Each pairing represented an association between the two (target and attribute) categories, and faster responses were indicative of a stronger association. For example, the target category of “me” might be paired in two blocks with the attribute category of “drinker” on the left of the screen, and “not me” with “non-drinker” on the right of the screen. In other blocks, this would then switch to “me” and “non-drinker” on the left and “not me” and “drinker” on the right. Faster reaction times in the blocks where “me” and “drinker” or “not me” and “non-drinker” were combined indicated a stronger association with these categories. Counterbalancing of blocks took place. The eventual IAT score of interest, the D-score (D-Biep), represented the standardized difference in average response time, and a higher score indicated faster response times when pairing “drinker” and “me.” Adhering to recommendations in earlier research, IAT scores were excluded from analyses when individuals committed errors on more than 30% of the trials, or when participants completed 10% or more of the trials in 300 ms (33–35).

##### Obsessive Compulsive Drinking Scale (OCDS) and Alcohol Urge Questionnaire (AUQ)

The OCDS and AUQ are two questionnaires, used to assess experiences of craving [for the OCDS: (36, 37); for the AUQ: (38)].

#### Measures for the Identification/Severity of Mental Disorders

##### The M.I.N.I. International Neuropsychiatric Interview-Plus

The M.I.N.I. is a structured interview which measures the major psychiatric disorders as represented in the Diagnostic Manual for Mental Disorders. Validity and reliability measures point to sufficient values (39–42). Current diagnoses (or lack of them) were checked for DD and HC groups by assessment of the M.I.N.I. and by reading the patient files concerning diagnostic procedures that were followed earlier.

##### European Addiction Severity Index (EuropASI)

The interview was used to check the presence and severity of current and past Substance Use Disorders (43, 44). Furthermore, this interview measures problems in several life domains.

##### Alcohol Use Disorder Identification Test (AUDIT)

The AUDIT was developed and approved by the World Health Organization (WHO) as a self-rating measure to identify risky/damaging patterns of alcohol use, and it may also be seen as a measure indicating severity of addictive (alcohol drinking) behavior [(45, 46), Dutch translation by Schippers and Broekman (47)]. As outlined, most patients (23) used alcohol as a major part of their SUD, and one of the male patients that did not have a current disorder in alcohol use, did come into trouble with alcohol use in his past.

### Procedure

Prior to the start of the study, approval for a broader neuropsychological project concerning self-regulation, where this study is a substantial part of, was obtained from the Radboud University Nijmegen Faculty of Social Sciences and its Ethics Committee for Behavioral Scientific Research (ECG; Protocol number ECSW2013-1811-148, letter number OOM/MB/13U.016587) and from the Vincent van Gogh Institutional Review Board (CWOP; Protocol U12.046). The research was performed in accordance with the principles of the declaration of Helsinki. Participants with DD were recruited by informing therapists in several departments of Vincent van Gogh Institute for Psychiatry (like addiction care and a department for Dual Disorders) about the scientific research. Consequently, patients, mostly hospitalized or in a 3 days a week ambulatory therapy program, were asked for voluntary participation by the main researcher. Freedom of choice to participate or refuse in this research was protected by securing absence of a therapist position of the main researcher to the patient that was asked. The advantage of primarily asking patients that were hospitalized or in a training program for several days a week, was that substance abstinence was regularly monitored by urine alcohol/drug testing. Healthy control group participants were recruited by means of social networking inside and outside the work institution; no psychologists were recruited, to prevent knowledge of the tasks of use in this study. Participants were provided with an information brochure detailing the study and consent form, which they read and signed. All assessed DD and HC participants were informed that they would afterwards receive a correspondence containing a broad descriptive strengths-and-weaknesses report of their results. Participants were selected on the basis of strict inclusion and exclusion criteria as previously outlined (see participants section). As a consequence of this, several DD and HC group participant data were eventually excluded from analyses, when exclusion criteria came to light on the assessment day itself (during the thorough assessments by the earlier mentioned M.I.N.I.-plus and EuropASI interviews), or when (even after succeeding test participation) any substantial hesitations were present concerning the reliability of patient's abstinence periods. Furthermore, exclusions from data analyses took place when patients showed error numbers or response patterns that were signs of non-validity. Consequently, data of 30 patients in the DD group were eventually excluded: seven patients dropped out, for example, because of flu and lack of motivation to plan another appointment; nine sets of patient data were excluded because of lower-than-expected intelligence scores; for five patients neurological incidents in their history came to light during the interviews; of three patients a (not mentioned) lack of substance abstinence was revealed after testing took place by regular treatment urine samples; and six patients showed invalid/non-explainable extreme outlier profiles or missing data on questionnaires or neurocognitive tasks. The occurrence of (extreme) outliers was checked, by constructing box plots for the DD, HC and total group's data. Data were only excluded from analyses when the extreme outlier was interpreted as non-explainable/non-realistic (e.g., a high composite craving, or error score on base of several tasks may be realistic when no sign is present that the participant misunderstood the instruction and response form, or gave random responses on tasks, and did not fall within levels of non-valid error/reaction time-rates on tasks). For the HC group, data sets were excluded when present psychiatric disorders or problematic substance use (in past or present) came to light during testing day interviews (two participants), or when invalid/incomplete questionnaires or missing tasks were present (four). Eventually, valid data of 25 DD patients remained for analyzes and the number of assessed HC group participants was adjusted to that. Thus, 25 patients with DD and 25 HC fully met inclusion criteria and showed valid test results. Concerning the ERP data, 15 valid DD group data (12 males, 3 females) and 18 valid HC group data (7 males, 11 females) remained for analyses after artifact reduction took place. All participants were tested individually in a well-lit and undisturbed room which is part of the Center of Excellence for Neuropsychiatry, Vincent van Gogh Institute for Psychiatry; this location also included an ERP lab room. All tests were administered and scored, adhering to official test instruction manuals.

#### ERP Recording and Data Reduction

ERP data were recorded adhering to official procedures, and analyzed in accordance with earlier studies involving aspects of inhibitory control (N200 and P300). Thirty two electrode sites and an acti-CAP (Brain Products) with active Ag/AgCI-electrodes were used for the ERP recordings. Besides the ground and reference electrode sites, the following electrode sites were used: Fp1, Fp2, F7, F3, Fz, F4, F8, FC5, FC1 (Heog 1), FCz (Reference), FC2 (Heog 2), FC6, T7, C3, Cz, C4, T8, Tp9, Cp5, Cp1 (Veog), Cp2 (Veog), Cp6, Tp10 (right mastoid), P7, P3, Pz, P4, P8, PO9, O1, Oz, O2, PO10. All signals were digitalized with a sample rate of 500 Hz and 24 bit A/D conversion. Subsequently, the following analysis steps were followed: re-referencing of the data, filtering, segmentation, artifact reduction, baseline correction, calculation of subject and grand averages, and consequently the statistical analysis of data (48). Data were re-referenced offline to the right mastoid (as used regularly in ERP research). Electroencephalographic (EEG) activity was filtered with band pass of 0.10–30 Hz (phase shift-free Butterworth filters; 24 dB/octave slope). Segmentation took place in epochs of 1 s (200 milliseconds before and 800 milliseconds after the stimulus response). For ocular correction, the Gratton technique was used (49); epochs including an EEG signal exceeding ±75 uV were excluded from the average. As a baseline, the 100 ms pre-response period served. After baseline correction, average ERP waves were calculated for artifact-free trials at each electrode site for correct responses. As described earlier, the N200 was defined as the mean amplitude value in the 200–300 ms time segment after onset of the response. The P300 wave was defined as the mean amplitude value in the 300–450 ms time segment after onset of the response. Segments that contained incorrect responses were excluded from analyses (Go trails with incorrect responses, or No-Go trails with false alarms). As said, 15 valid DD group data (12 males, 3 females) and 18 valid HC group data (7 males, 11 females) remained for analyses after artifact reduction took place.

#### Data Analysis

SPSS version 25.0 was used for the statistical analyses. MANOVAs with bootstrapping and Bonferroni corrections (for multiple comparisons) were conducted to compare (composite) score differences between the DD and HC groups for inhibitory control and craving (50). Sex was used as an extra between-subjects factor in analyses. Repeated-Measures-ANOVAs (RM-ANOVAs) (with contrasts) were conducted to analyze performance outcomes on the Go/No-Go, and ERP inhibitory control indices. Between-subjects factors in RM-ANOVAs were group (DD vs. HC) and sex (male vs. female). Two-level within-subject factors were taken into account, namely Group × Inhibition (Go or No-Go) × Drink (Alcoholic or Non-alcoholic). For ERP analyzes three-level within subject factors were taken into account (Group × Inhibition × drink × Electrode). Lastly, Spearman correlation coefficients were calculated in order to analyze the relations between the concepts of inhibitory control and craving, and on exploratory base, binary logistic regression analysis was performed to check the predictive value of these concepts for the classified severity of addictive behavior (two categories of dependent variable severity of addictive behavior: a score of 14 or less, vs. a score of 15 or more on the AUDIT.

## Results

Main findings are presented for the DD group (*n* = 25) vs. the HC group (*n* = 25) concerning neurobehavioral and self-rating measures of inhibitory control, and measures of craving. Next, the ANOVA data for brain ERP measures of inhibitory control are presented. When a significant group × sex interaction effect is present, then group and sex findings are presented separately. Estimated marginal means and standard errors for the groups are presented. Finally, correlational and regression analyses are presented. For clarity, results from all analyzes are visualized by separate Tables and Figures.

### Inhibitory Control and Craving

For group (DD vs. HC, see Table 4), a significant main effect was present on composite score measures of inhibitory control and craving, indicating that DD patients showed more problems in one or more of these domains than HC, *F*_(8, 39)_ = 3.61, *p* < 0.01, Partial η^2^ = 0.43). No significant main effect of sex, or Group × sex interaction effect was present.

**Table 4:** Dual disorders vs. healthy control group composite scores and subscale scores comparisons with significance levels [(**p = 0.01; *p = 0.05); estimated marginal means, standard errors in parentheses].

**Disorderddaranii scale**	**Dual disorders** (*n* **= 25)**	**Healthy** **controls** (*n* **= 25)**	**Significance** **(*p*.)**
Inhibitory control total commission errors No-go	3.94 (0.65)	1.86 (0.58)	0.02^*^
Inhibitory control commission errors beer No-go	2.54 (0.44)	1.22 (0.40)	0.03^*^
Inhibitory control commission errors juice No-go	1.40 (0.32)	0.65 (0.29)	0.08
Mean reaction time Beer Go condition	393.96 (10.56)	391.85 (9.40)	0.88
Stop Signal Task proportion successful stops	0.53 (0.03)	0.50 (0.02)	0.35
Impulsivity BIS-11 composite score	69.62 (2.86)	54.39 (2.54)	<0.01^**^
Craving OCDS and AUQ composite score	12.82 (2.19)	0.59 (1.95)	<0.01^**^
Craving implicit measure IAT D-Biep	0.05 (0.14)	−0.32 (0.12)	0.05^*^

The results demonstrated that DD patients had more inhibitory control problems than HC, in the form of a higher amount of commission errors on the (low frequent stimulus) No-go condition of the consumption related Go/No-go task [*F*_(1, 46)_ = 5.77, *p* = 0.02, Partial η^2^ = 0.11]. And, in relation to specifically alcohol No-go trial pictures this DD-HC group difference in commission errors was present as well [*F*_(1, 46)_ = 4.94, *p* = 0.03, Partial η^2^ = 0.10]. Moreover, patients with DD reported higher amounts of impulsivity, and higher levels of explicit and implicit alcohol craving than HC, as observed in composite scores (respectively *F*_(1, 46)_ = 15.84, *p* < 0.01, Partial η^2^= 0.26; *F*_(1, 46)_ = 17.46, *p* < 0.01, Partial η^2^ = 0.28; *F*_(1, 46)_ = 3.96, *p* =0.05, Partial η^2^ =0.08). In contrast, no significant group differences were present for the number of errors committed on a non-consumption related inhibitory control task, and for reaction times on the Go/No-go task [respectively *F*_(1, 46)_ = 0.89, *p* = 0.35, Partial η^2^= 0.02; *F*_(1, 46)_ = 0.02, *p* = 0.88, Partial η^2^ < 0.01].

### ERP Data: P300 Component

**Figure 2:**
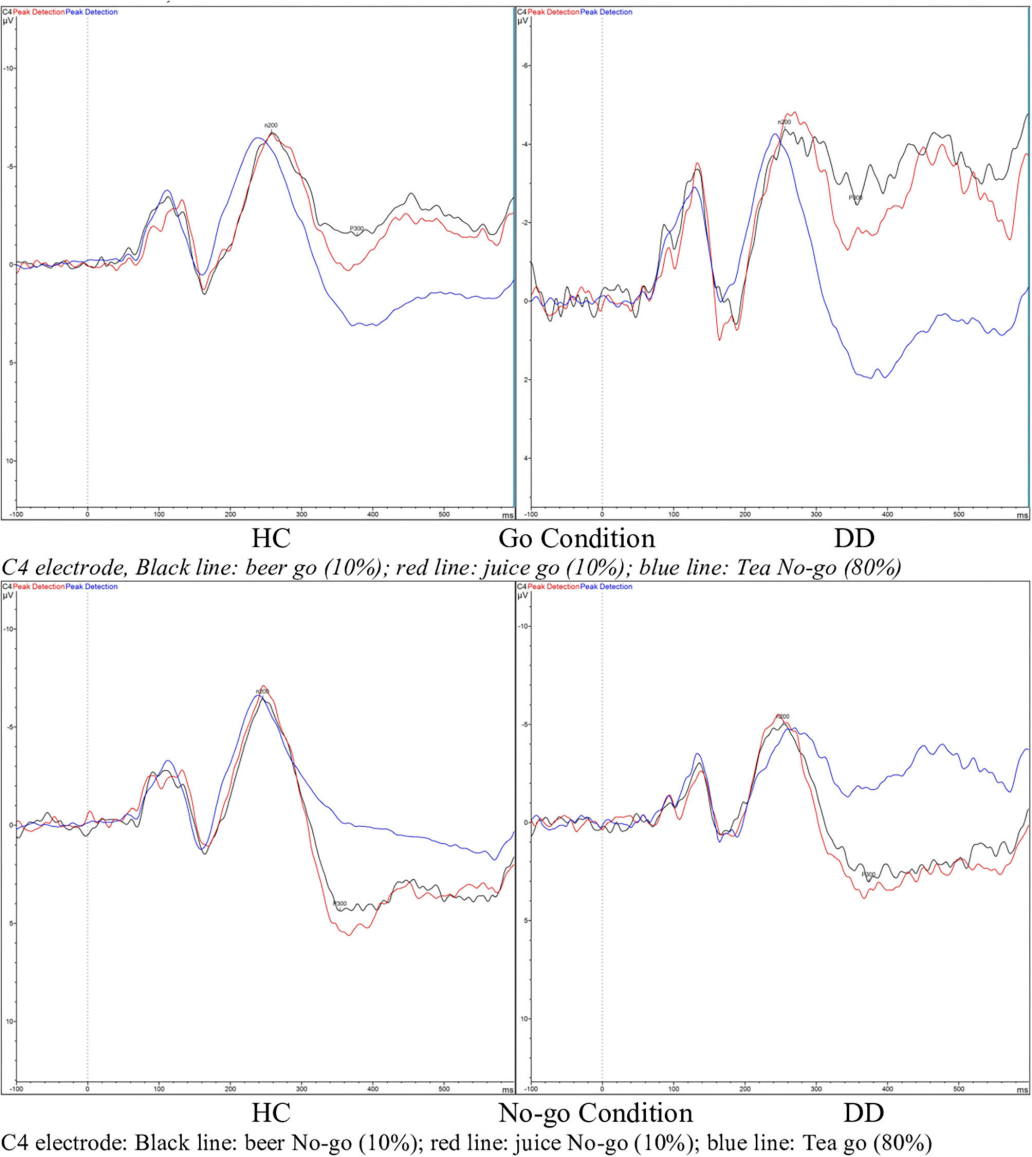
Event related potential (ERP) graphs of P300 and N200 components for DD patients and healthy controls on a Go/No-go task, with alcohol-related and non-alcohol related stimuli, electrode C4.

**Figure 3:**
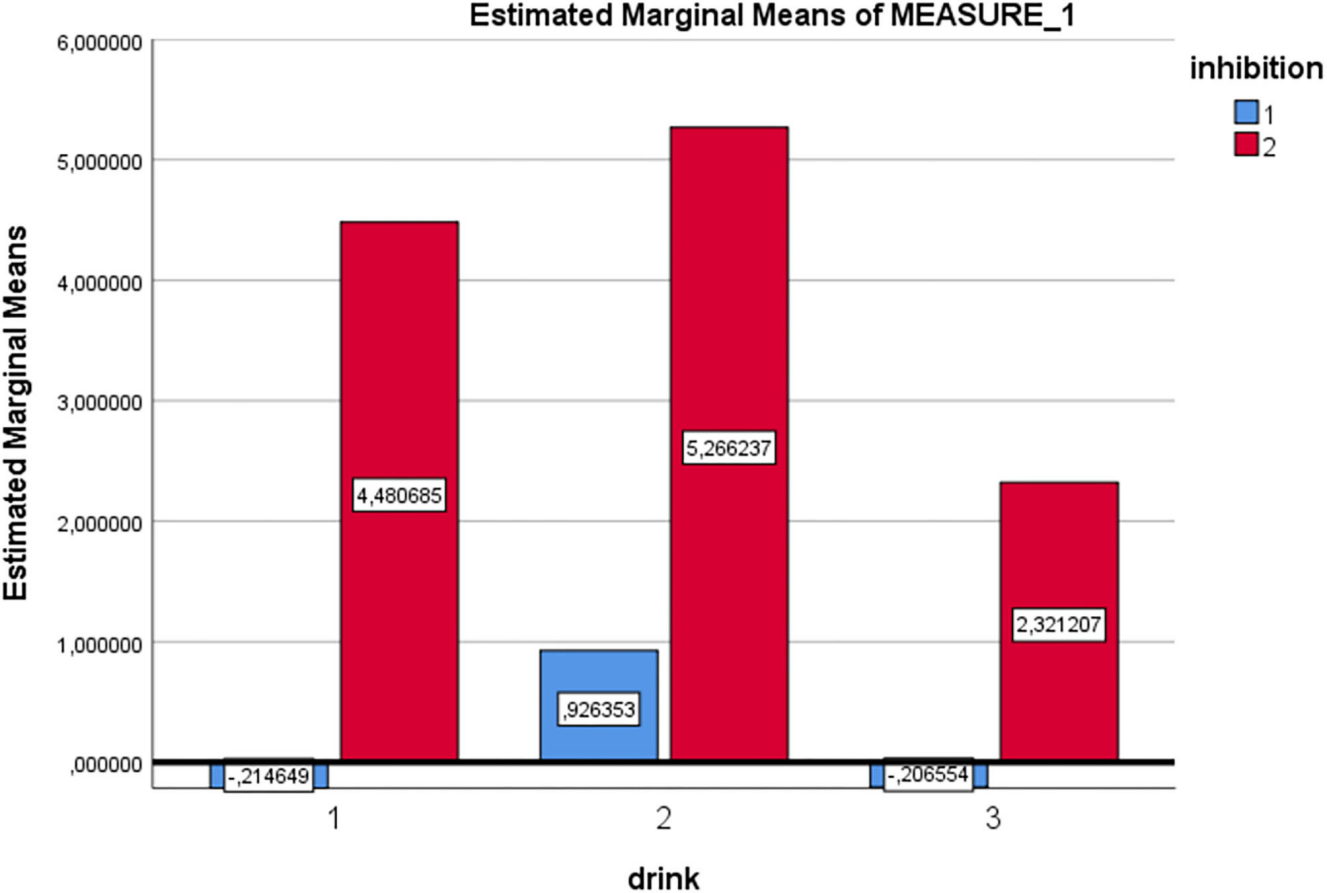
Estimated marginal mean P300 amplitude values [Drink 1 = beer (low frequent), drink 2 = juice (low frequent), drink 3 = tea (high frequent), inhibition condition 1 = Go, inhibition condition 2 = No-Go (for all drinks in this graph)].

RM-ANOVA analysis of the Go/No-go task P300 ERP component measure revealed a main effect for Inhibition (Go/No-go). Thus, the expected P300 amplitude peak actually occurred within the 300–450 millisecond post-stimulus No-go area as a reaction to the low frequency stimulus that required inhibitory control [see Figures 2, 3 and Table 5; *F*_(1, 29)_ = 60.75, *p* = < 0.01, Partial η^2^= 0.68)]. Moreover, a main drink x group x sex interaction effect was present [*F*_(2, 28)_= 3.89, *p* = 0.03, Partial η^2^ = 0.22]. That is, female DD patients showed reduced mean P300 amplitudes in reaction to alcohol pictures, when compared to female HC patients; however, this effect involves ERP data for which only three female DD data were present. Therefore, this effect was not further evaluated here. Furthermore, a main effect for drink, and an inhibition x drink interaction effect were present for the P300 component. That is, both patients with DD and HC showed reduced mean P300 amplitude in overall response (independent of Go/No-go conditions) to non-frequent alcohol related stimuli appearances (beer pictures) vs. non-frequent non-alcohol related stimuli appearances (juice pictures) [see Figures 2, 3 and Table 5; *F*_(2, 28)_ = 8.13, *p* = < 0.01, Partial η^2^= 0.37]. Furthermore, the differences between mean P300 amplitudes on No-go and Go conditions were higher for both patients with DD, and HC when responding to the non-frequent stimuli, as compared to responses on No-go and Go conditions to high-frequency (non-alcohol) related stimuli (tea pictures) [see Figures 2, 3 and Table 5; *F*_(2, 28)_= 4.37, *p* = 0.02, Partial η^2^= 0.24]. Finally, there was a main effect for the interaction of drink x inhibition x electrode x sex [*F*_(6, 24)_ = 3.33, *p*= 0.02, Partial η^2^= 0.45].

**Table 5:** N200 and P300 component estimated marginal mean amplitudes for DD patients and HC.

		**Controls**	**Controls**	**Patients**	**Patients**
		**Fz**	**C4**	**Fz**	**C4**
**Estimated marginal mean Amplitude value (estimated marginal mean, standard error in parentheses in uV)**					
N200	Beer Go	−8.47 (1.08)	−8.34 (0.93)	−7.60 (1.44)	−6.81 (1.24)
	Juice Go	−8.08 (1.38)	−7.86 (1.16)	−7.81 (1.85)	−7.43 (1.54)
	Tea Go	−8.11 (1.17)	−7.51 (0.99)	−6.40 (1.56)	−5.64 (1.33)
	Beer No-go	−9.10 (1.44)	−7.94 (1.22)	−7.32 (1.92)	−7.00 (1.63)
	Juice No-go	−9.37 (1.51)	−8.41 (1.24)	−8.13 (2.01)	−7.47 (1.66)
	Tea No-go	−8.13 (1.22)	−7.11 (1.04)	−6.45 (1.63)	−4.74 (1.38)
P300	Beer Go	0.21 (1.16)	0.65 (1.20)	−1.44 (1.55)	−2.24 (1.61)
	Juice Go	1.33 (1.11)	1.74 (1.03)	0.16 (1.48)	−0.63 (1.38)
	Tea Go	−0.23 (0.83)	1.26 (0.90)	−0.81 (1.11)	−0.25 (1.20)
	Beer No-go	6.10 (1.46)	6.63 (1.12)	4.49 (1.95)	3.00 (1.50)
	Juice No-go	7.05 (1.61)	7.56 (1.19)	5.20 (2.15)	3.95 (1.59)
	Tea No-go	3.38 (0.97)	4.03 (0.72)	2.48 (1.30)	2.20 (0.96)

### ERP Data: N200 Component

On RM-ANOVA analysis of the Go/No-go and N200 component ERP measure, no main group/sex/group × sex/other interaction effects were revealed at all. Therefore, no inhibition (Go/No-go) difference was present either, which indicates that the mean peak values of the N200, occurring in the 200–300 post-stimulus area in reaction on a low frequent stimulus that required execution of inhibitory control, did not differ from the mean amplitude values of the N200 in the 200–300 post-stimulus area as a reaction on a low frequent stimulus that did not require execution of inhibitory control [see Figure 2 and Table 5; *F*_(1, 29)_ = 1.43, *p* = 0.24, Partial η^2^= 0.05]. However, there was a main effect for drink; in reaction to low frequent pictures of juice, the mean N200 amplitude value was higher (more negative) than the mean N200 amplitude in a reaction to high frequent tea pictures [main effect data: *F*_(2, 28)_ = 3.41, *p* = 0.05, Partial η^2^= 0.20; juice vs. tea data: *F*_(1, 29)_= 6.83, *p* = 0.01, Partial η^2^= 0.19]. This difference was not present when comparing the mean N200 amplitudes of low frequent pictures of alcohol and high frequent tea pictures.

### Relational Findings

**Figure 4:**
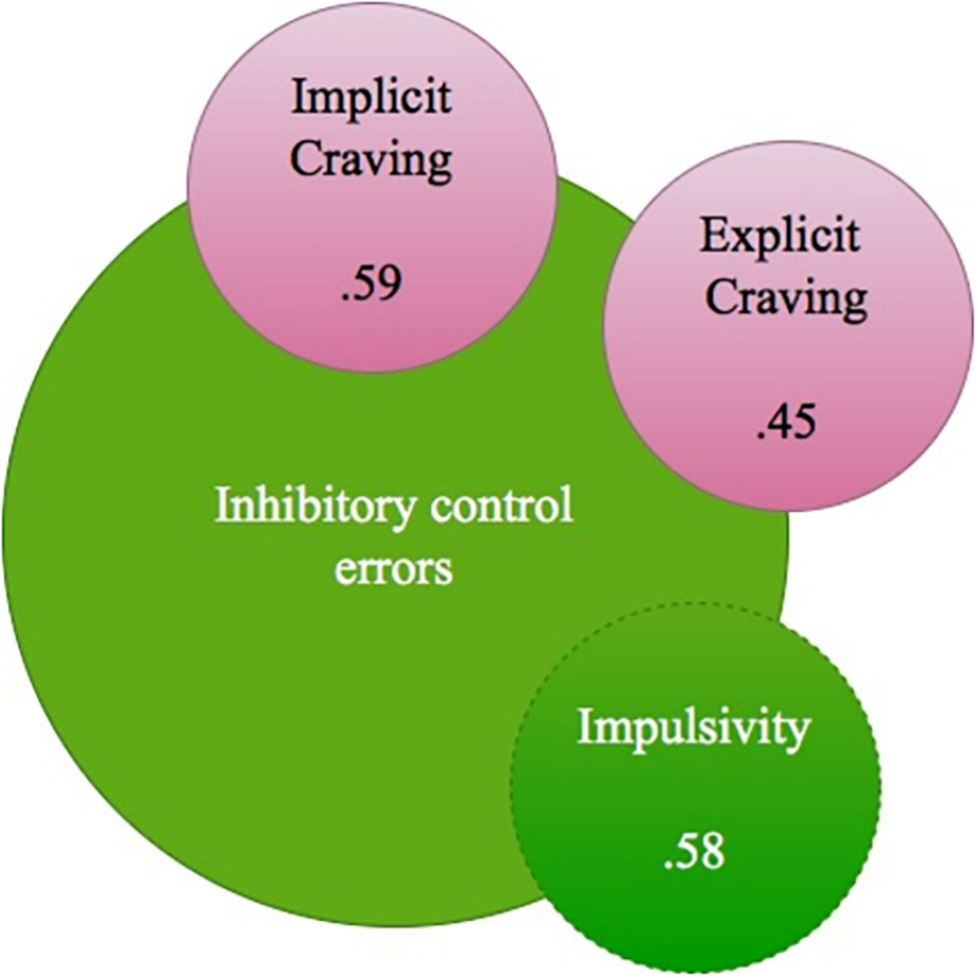
DD group relations between Inhibitory control errors, impulsivity and (implicit and explicit) craving (Spearman's rho's).

When evaluating the DD group (*n* = 25) and relations between inhibitory control and craving, a positive relation was present between the amount of inhibitory control errors on the Go/No-go task and the level of self-reported impulsivity as part of inhibitory control (Spearman's rho 0.58^**^), and both explicitly and implicitly measured craving (Spearman's rho's respectively 0.45^*^ and 0.59^**^). Higher amounts of inhibitory errors were related to reduced levels of mean P300 Beer-Go amplitude (for the C4 electrode, Spearman's rho −0.52^*^). A higher amount of craving was related to reduced levels of mean N200 Beer Go and mean N200 Jus Go amplitudes, most pronounced at the F3 electrode (Spearman's rho's 0.73^**^ and 0.54^*^) (For findings also see Figure 4).

### Binary Logistic Regression Analysis

Finally, an exploratory binary logistic regression analysis was conducted for the whole group of participants, with the severity of addictive drinking behavior (AUDIT) as the dependent variable. Due to the low participant sample size (*N* = 50) a minimum of predictor variables was strived for. Therefore, initially the following independent variables were put hierarchically into the predictive model to test their contributive significance: impulsivity level, amount of inhibitory errors on the Beer no-go stimuli (Go/No-go task), level of explicit craving (composite craving score OCDS and AUQ), level of implicit craving (D-biep score IAT) and the interaction of impulsivity level and explicit craving. The level of implicit craving variable and the interaction of explicit craving and impulsivity did not reveal significant contributions to the model independently and were therefore excluded from the model. Furthermore, tests for linearity of logits and multicollinearity were undertaken and revealed no issues. Eventually, the model with three predictor variables revealed a Chi^2^ of 19.07 (*p* < 0.01), and the predictors accounted for 43.9 % of the variance in outcome (Nagelkerke *R*^2^). See Table 6 for an overview of findings. Summarizing, impulsivity and the level of explicit craving were of independent predictive value for a higher severity of addictive drinking behavior.

**Table 6:** Logistic regression analysis coefficients of the model, concepts predicting whether a participant showed a score of 15 or higher on the AUDIT (severity addictive drinking behavior).

**Step 1**	**Wald**	**b**	**SE β**	**Exp. (β)**	***P***	**95% confidence interval for Exp. (β)**	
Constant	9.51	−6.27	2.03	<0.01	<0.01^**^		
Impulsivity	7.29	0.09	0.03	1.09	0.01^**^	1.03	1.17
Explicit craving	4.17	0.14	0.07	1.15	0.04^*^	1.01	1.31
Inhibitory errors beer No-go	3.23	−0.52	0.29	0.59	0.07	0.34	1.05

R^2^ = 0.32 (Cox and Snell R Square), 0.44 (Nagelkerke R Square), Chi^2^ (3) = 19.07 (Omnibus Tests of model coefficients), p < 0.01, Chi^2^(8) = 10.48, (Hosmer and Lemeshow test) p = 0.23. Significance levels are stated as [(**p = 0.01; *p = 0.05)].

## Discussion

### Aim and Main Question

This brain-behavior study aimed to contribute to the understanding of the recurrent addictive behavior in patients with DD, by zooming into the neurocognitive function of inhibitory control and into craving from a multiple method perspective. For this purpose, both electrophysiological, neurobehavioral and self-report measures were conducted within a DD patient and HC group. The main question was, whether patients with DD, suffering from recurrent substance use, demonstrate impaired inhibitory control and/or higher levels of craving, as compared to HC. Moreover, as a sub-question: are the levels of inhibitory control and craving interrelated, possibly also strengthening the severity of addictive (drinking) behavior?

### Findings

As suggested in the first hypothesis concerning neurobehavioral inhibitory control and craving comparisons between patients with DD and HC, patients with DD did indeed commit more inhibitory control errors than HC on a Go/No-go task that involved consumption stimuli, and this was the case for alcohol pictures specifically. Interestingly, the finding solely occurred on this consumption related task but not on a non-consumption related stop signal task, and only when low frequent No-go picture stimuli were present in the context of high frequent Go picture stimuli. This finding may indicate that inhibitory control impairments of patients with DD are limited to substance-cue situations (like alcoholic stimuli), maybe specifically when these stimuli occur rather unexpectedly within the context. Whereas substance-abstinent DD patients may in general situations have sufficient inhibitory abilities. The second hypothesis, which expected N200 and P300 ERP data to reveal DD and HC differences in the form of reduced amplitudes in patients with DD, was not confirmed. ERP analyses did not indicate differences in inhibitory control-associated brain activity between DD patients and HC; both groups illustrated a reduction of P300 amplitudes in reaction to alcohol pictures. The third hypothesis was confirmed; that is, levels of implicit and explicit craving were both positively associated with the amount of errors in inhibitory control. This fits the literature that described the neurobiological interrelatedness of craving and inhibitory control in addiction (4). The fourth hypothesis stated that the interaction of inhibitory control and craving would demonstrate a predictive value for the classified severity of addictive (drinking) behavior. Thus: the more inhibitory control errors one commits, and the more craving one experiences, the more severe one's daily life addictive (drinking) behavior will be (as classified on a substance use disorder identification test). This hypothesis, via exploratory analysis due to the low participant sample size, was not confirmed. Explicit craving level and impulsivity (as part of inhibitory control) did indicate independent predictive values for the severity level of addictive behavior, but implicit craving level and the interaction of explicit craving and impulsivity did not reveal such predictive values.

The findings as demonstrated throughout this study do correspond with past research, including earlier developed addiction/craving models of Koob and Volkow (4) and Field and Cox (11). Relations between impaired inhibitory control and craving were confirmed, and specifically for consumer cues. This may also explain the lack of differences between the DD and HC group on a non-consumption related task in the present study. A recent large review concerning inhibitory control in (mostly recreational) substance use also revealed that inhibitory control is not per definition “overall” impaired for this behavior. And, when studying concepts, it is always necessary to be alert for potential confounders of findings. For example, sex differences between groups may influence results on measures of main interest (51).

Contributive of the present studies' findings to the existing knowledge concerning Dual Disorders and its recurrent substance use behavior, is that patients with DD demonstrate more impulsive tendency and impaired inhibitory control when confronted with consumption related stimuli, as compared to HC. Further, levels of inhibitory control errors and craving are positively related. Moreover, the level of craving that is consciously experienced and impulsivity levels are of independent predictive value for the severity of addictive drinking behavior.

Electrophysiological (brain) data concerning the P300 and N200 ERP components did partly fit earlier data, as described in research studies and reviews from, among others, Luijten et al. (15). That is, the P300 did actually occur within the expected time area and expected P300 amplitude differences were present for responses to high-frequent and low-frequent stimuli, low-frequent stimuli evoking higher mean amplitudes [among others, in (16)]. But, there were no evident group differences with respect to the P300 and N200 components. The reduction in P300 amplitude is, as mentioned earlier, frequently associated with impaired inhibitory control. But, contrary to expectations, these reduced P300 amplitudes were present in response to alcohol related pictures for both Go and No-go trials, and in both the DD and HC group. In past craving research, larger ERP P300 components were signaled for patients with alcohol use disorders on alcohol related stimuli tasks. However, this was not revealed in the present study. The absence of a group effect could be explained by the fact that all patients with DD that participated were in (mostly intensive 24–7 or 3 days a week) treatment for their substance use disorders and were abstinent for substantial numbers of weeks (range 6 to 32 weeks). Thus, one possible explanation is that these patients with DD have already learned how to deal with thoughts of craving/show less differing P300 components as compared to HC. Elaborating on this, it may be a recommendation for studying the P300 component in the Go/No-go task within a DD group that is abstinent for a shorter period of time.

### Limitations

Several limitations of this study need to be considered. The first major one concerns the effect sizes and sample sizes that were small (25 DD patients and 25 HC), when looking at the amount of variables taken into account and amount of analyses as undertaken. (Sub) group comparisons were underpowered due to this limitation and therefore were not included in this paper. As a consequence of all of this, conclusions should be taken cautiously and further research in larger patient groups is required. However, the execution of this study emphasizes the importance of clinical practice research, even when the gain of participants is a challenge and takes time and effort.

An additional limitation of the current study is the use of purely alcohol (and non-alcohol) related stimuli in the Go/No-go/IAT tasks and severity of addictive behavior measure (AUDIT) instead of a combination of alcohol and other drug related stimuli. However, for the majority of patients with DD alcohol was part of the substance use disorder (23 of 25 patients with DD, and 1 of the two male patients that only used drugs came into trouble with alcohol use earlier in his life). The decision for the use of the AUDIT score as estimate of the severity of addictive behavior, instead of the EuropASI interview score concerning the severity of substance use problems, was that the latter score is determined by the clinical review of the researcher that administers the task. In order to prevent any influence of biases in this research, the AUDIT score was used, which is fully determined by the answers the participant gives.

Finally, a significant sex difference that was present between the DD and HC groups resulted in a dilemma during ERP P300 data interpretation. Due to the limited number of valid female DD group data (3), this effect could not be accurately analyzed in order to draw conclusions from it. It deserves further research to perform comparisons between male and female DD patients on ERP measures (of inhibitory control in reaction to alcohol/non-alcohol related stimuli).

### Future Research

Elaborating on the findings that impulsivity and craving have predictive value for a higher severity of addictive behavior, one could suggest that, when one tends to be more impulsive and urging, one regularly may want immediate gratification of desires like rewards or want alleviation of frustrations (52). That is, the tolerance for frustrations of impulsive and/or craving persons may be somewhat lower, triggering more (severe) recurrent substance use behavior to feel better. In light of this, a recent review from Sliedrecht et al. (53) revealed that the factor of gratification of desires is of minor influence on relapses in alcohol related behavior, whereas the urge for relief from unpleasant emotions is of more influence. Further research is required to test this suggestion concerning the relation between impulsivity, the urge for alleviation from frustrations and the severity of substance use behavior in a DD group. Furthermore, following up this study, Beerten-Duijkers et al. (54, 55) translated and adapted the Barkley Deficits in Executive Functioning Scales (BDEFS) for the Dutch language clinical practice. This self-rating questionnaire measures inhibitory control and impulsive tendencies as well as emotion regulation in reaction to potentially frustrating situations. The BDEFS can thereby contribute to clarification of the relations between impulsive tendencies, emotion/frustration regulation and craving.

Furthermore, the gain of longitudinal data concerning recurrent substance use (e.g., number of relapses) leads to findings that link more to the real-world measure of inhibitory control impairments. That is, it would be interesting to see if the present findings, like the predictive value of impulsivity and explicit craving would also be translated in a higher level of relapses in addiction related behaviors.

Furthermore, the time of day at which a participant is tested can be of influence on functioning and therefore needs to be taken into account in future research (56). In the current research, the time of day was flexible, depending on the preference of participants in order to promote willingness to cooperate and to prevent drop outs.

### Clinical Implications

Overall, this study's findings promote a better neuropsychological understanding of recurrent substance use in DD patients by measuring inhibitory control and craving. On the basis of the findings, additional attention is recommended for impulse control training within this patient group (training focused on Stop-Think-Act strategies). Training in this area may promote effective coping strategies for patients with DD who are particularly sensitive regarding impulsive tendencies that may eventually lead to severe substance abuse behavior. In currently available evidence-based treatment programs for addiction groups, craving is usually integrated already [for instance, in the Approach-avoidance training; (57, 58)]. However, as explained, a differentiated insight in one's functioning on addiction underlying concepts like inhibitory control and craving is of contributive importance for the understanding of one's strengths and pitfalls, next to more topographic knowledge concerning disorder classifications. When solely taking the latter as the lead, more standardized training accents might be used for a patient group as a whole, instead of keeping sight on one's specific needs in certain domains. An integrative view from the brain-behavior perspective transcends the topographic diagnostic classification knowledge and may thereby truly form a key contribution on the path to the stepwise prevention of recurrent addictive behavior.

## Data Availability Statement

The raw data supporting the conclusions of this article will be made available by the authors, without undue reservation.

## Ethics Statement

The studies involving human participants were reviewed and approved by Ethics Committee for Behavioral Scientific Research (ECG), protocol number ECSW2013-1811-148. The patients/participants provided their written informed consent to participate in this study.

## Author Contributions

JB-D performed the literature search, coordinated the data collection/analyzes and drafted the manuscript, together with CV. In addition, CV, MR, and JE were available for consultation and discussion during the project and during multiple rounds, revised the manuscript for important content. All authors contributed to the article and approved the submitted version.

## Conflict of Interest

The authors declare that the research was conducted in the absence of any commercial or financial relationships that could be construed as a potential conflict of interest.

## Abbreviations

DD: dual disorders
ERP: event related potentials
HC: healthy controls
SUD: substance use disorder.
